# Comparative Peripheral Blood Transcriptomic Profiles in Parkinson’s Disease and Restless Legs Syndrome: An Exploratory Between-Cohort Analysis

**DOI:** 10.3390/ijms27156576

**Published:** 2026-07-23

**Authors:** Maria P. Mogavero, Giovanna Marchese, Giovanna M. Ventola, Giuseppe Lanza, Stefan Clemens, Michele Salemi, Oliviero Bruni, Raffaele Ferri

**Affiliations:** 1 Oasi Research Institute-IRCCS, 94018 Troina, Italy; paola_mogavero@libero.it (M.P.M.); glanza@oasi.en.it (G.L.); msalemi@oasi.en.it (M.S.); 2 Genomix4Life Srl, 84081 Baronissi, Italy; giovanna.marchese@genomix4life.com (G.M.); giovanna.ventola@genomix4life.com (G.M.V.); 3 Genome Research Center for Health (CRGS), 84081 Baronissi, Italy; 4 Department of General Surgery and Medical-Surgical Specialties, University of Catania, 95123 Catania, Italy; 5 Department of Physiology, Brody School of Medicine, East Carolina University, Greenville, NC 27834, USA; clemenss@ecu.edu; 6 Department of Human Neuroscience, Sapienza University of Rome, 00185 Rome, Italy; oliviero.bruni@uniroma1.it

**Keywords:** restless legs syndrome, Parkinson’s disease, peripheral blood transcriptomics, dopaminergic pathways, sleep-related movement disorders

## Abstract

Restless legs syndrome (RLS) and RLS-like symptoms are frequently reported in Parkinson’s disease (PD), but whether this clinical overlap reflects shared biology or convergent symptom expression remains uncertain. We performed an exploratory comparative analysis of peripheral blood mononuclear cell RNA-seq data from 17 drug-free patients with RLS and 60 patients with PD, most of whom were receiving dopaminergic treatment. Because the two cohorts were derived from independent datasets and no healthy control group was included, all findings were interpreted as relative PD-versus-RLS differences rather than disease-specific alterations. Differential expression and pathway analyses identified broad transcriptomic separation between the two groups. Genes with relatively higher expression in PD than RLS included transcripts related to dopamine metabolism, tetrahydrobiopterin biology, iron transport, oxidative stress, and inflammatory signaling, whereas genes with relatively higher expression in RLS than PD involved iron/ferroptosis-related biology, GPCR-mediated signaling, synaptic modulation, and circadian-related pathways. These results suggest that peripheral blood transcriptomics may help generate hypotheses on the biological heterogeneity underlying RLS-like symptoms in PD. However, because of treatment imbalance, cohort differences, batch-group confounding, absence of healthy controls, and the use of PBMCs as a peripheral tissue, the findings should be considered hypothesis-generating and require independent validation.

## 1. Introduction

Restless legs syndrome (RLS) is a common sensorimotor disorder characterized by an urge to move the legs, usually accompanied by unpleasant sensations, that begins or worsens during rest, is relieved by movement, and shows a clear evening or nocturnal predominance [1]. The biological basis of RLS is increasingly recognized as multifactorial, involving disturbed brain iron homeostasis, dopaminergic dysfunction, and broader molecular mechanisms extending beyond a single neurotransmitter system [2,3,4,5]. Recent large-scale genetic studies have further reinforced the viewpoint that RLS reflects a complex biological architecture involving multiple susceptibility loci and pathways relevant to disease biology [6].

Parkinson’s disease (PD), the most common neurodegenerative movement disorder [7], is a growing global health burden [8], is strongly associated with sleep and circadian disturbances [9], and is frequently accompanied by RLS-like symptoms or comorbid RLS [10,11].

For PD research, the distinction between true comorbid RLS, RLS-like symptoms, sensory discomfort, akathisia, nocturnal motor restlessness, and treatment-related phenomena is clinically important but biologically unresolved [10,11,12]. These phenotypes may arise from partially overlapping dopaminergic mechanisms, from distinct non-motor PD pathways, or from coincidental comorbidity with primary RLS; importantly, available genetic evidence does not support a clear causal relationship or substantial shared genetic architecture between RLS and PD [13]. Clarifying this issue is relevant not only for diagnosis, but also for therapeutic decision-making, because dopaminergic agents can improve symptoms in both disorders [14,15,16] while also causing long-term complications, including impulse-control disorders in PD and augmentation in RLS [17,18,19,20]. A molecular comparison between PD and well-characterized RLS therefore provides an opportunity to determine whether clinical overlap reflects shared disease biology or convergent symptoms arising from distinct molecular systems [13,21,22,23,24,25].

This clinical overlap is of considerable interest because epidemiological and meta-analytic studies have suggested that RLS is more frequent in PD than in the general population, although estimates vary across cohorts and diagnostic approaches [26,27]. At the same time, genetic studies have not supported a clear causal relationship or substantial shared genetic architecture between RLS and PD, suggesting that apparent clinical convergence may coexist with important biological divergence [13].

Moreover, large-scale genetic studies suggest that extrinsic or environmental factors, rather than genetic predisposition alone, play a predominant role in the development of PD [8]; in this context, transcriptomic studies are especially valuable because they may capture gene–environment interactions and help identify dysregulated biological pathways, potential biomarkers, and therapeutic targets [28].

Transcriptomic approaches offer an opportunity to move beyond symptom-level overlap and to define the biological programs associated with each disorder. In RLS, blood-based RNA sequencing has recently identified marked dysregulation of pathways related to inflammation, mitophagy, oxidative stress, and ferroptosis [21], and subsequent work has further implicated circadian disruption and ferroptosis-related mechanisms in the molecular landscape of the disease [22]. In PD, transcriptomic studies in peripheral blood and the brain have demonstrated alterations involving immune signaling, long non-coding RNAs, neurodegeneration-related pathways, and partially convergent molecular signatures between blood and brain tissue [23,24,25]. More broadly, blood transcriptome-based studies have supported the potential utility of peripheral gene-expression profiling for biomarker development in PD, even if clinical translation remains challenging [29].

However, these lines of research have largely developed in parallel, and the molecular relationship between RLS and PD remains insufficiently defined. A direct comparison is particularly relevant because both disorders touch on dopaminergic mechanisms, iron-related biology, immune–redox pathways, and circadian regulation, yet they differ profoundly in clinical trajectory and in the presence or absence of overt neurodegeneration [3,11,13,30]. Moreover, neuroimaging studies suggest that different dopaminergic pathways may be preferentially involved in the two disorders, with the nigrostriatal pathway playing a well-established role in PD [31], whereas the mesolimbic dopaminergic pathway appears to be more prominently implicated in RLS [32,33]. Notably, alterations in regions linked to the mesolimbic reward system have also been described in PD in association with depression and pathological gambling [17,34].

There is currently no direct biological evidence demonstrating differential expression of the two main dopaminergic pathways in RLS and PD, despite the important clinical and therapeutic implications. Although the D2-like receptor family (D2, D3, and D4) is implicated in both conditions, it seems to exhibit distinct expression patterns between the two diseases, highlighting the need for further research, particularly on mesolimbic circuits, to better understand treatment modulation in patients with PD and comorbid RLS. In PD, dopamine receptors mainly undergo functional changes and compensatory upregulation, especially D2, in response to dopaminergic loss and treatment exposure [16]. In contrast, in RLS, D2-like receptors are associated with therapeutic effects, while alterations to the D1 receptor system may contribute to symptom worsening during chronic therapy, including augmentation phenomena [18,19,35].

Given the frequent co-occurrence of RLS and PD, as documented by clinical and epidemiological studies [12], the management of these comorbid conditions is often challenging, particularly because of complications that may arise during long-term treatment with dopamine agonists in both disorders. Consistent with these concerns, recent RLS guidelines recommend dopamine agonists only as second-line therapy [20].

In this context, we performed a direct blood transcriptomic comparison between PD and clinically well-characterized RLS. Rather than treating RLS merely as a separate sleep-related movement disorder, we used it as a biologically informative comparator for PD, with the aim of identifying molecular signatures that distinguish Parkinsonian neurodegenerative biology from RLS-related sensorimotor and dopaminergic dysfunction. We focused particularly on pathways relevant to PD pathophysiology, including dopaminergic neuronal identity, tetrahydrobiopterin biosynthesis, iron handling, ferroptosis, neuroinflammation, oxidative stress, and circadian regulation.

## 2. Results

Because the PD and RLS samples originated from independently generated RNA-sequencing datasets, diagnostic group was completely confounded with dataset/batch. Consequently, the present analysis cannot distinguish disease-related differences from technical effects. In addition, most participants with PD were receiving dopaminergic therapy, whereas the RLS cohort was drug-free. Treatment may have influenced the observed differences, particularly those involving dopamine-related and other treatment-sensitive pathways; however, their magnitude and direction cannot be determined. The findings should therefore be interpreted exclusively as an exploratory between-cohort transcriptomic contrast that may reflect diagnosis, treatment exposure, technical processing, cohort composition, or a combination of these factors. All genes and pathways are described only as relatively higher in one cohort than in the other and not as disease-specific abnormalities relative to a physiological baseline.

### 2.1. Relative Transcriptomic Profiles in the PD and RLS Cohorts

**Figure 1:**
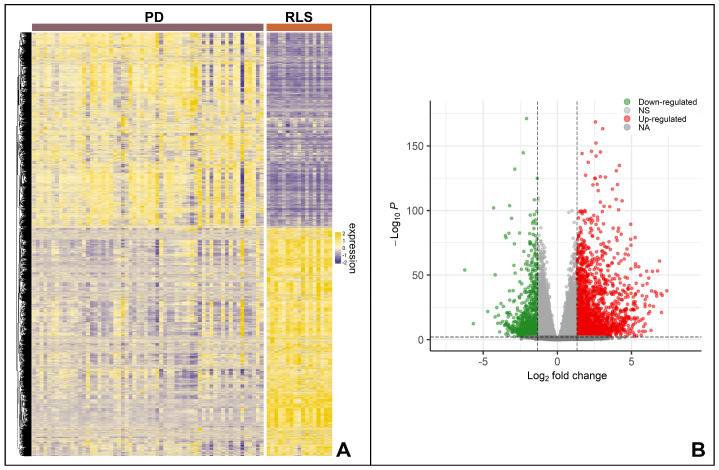
Global transcriptomic differences between the PD and RLS cohorts. (**A**) Heatmap of genes showing different expression levels between the two cohorts based on normalized gene-expression values. (**B**) Volcano plot highlighting genes with relatively higher expression in the RLS cohort and genes with relatively higher expression in the PD cohort according to the fold-change and statistical-significance thresholds. All directions refer exclusively to the RLS-versus-PD comparison.

Differential expression analysis comparing the RLS and PD cohorts, using a threshold of adjusted *p*-value ≤ 0.01 and |FC| ≥ 1.50, identified 7872 genes with different expression levels between the two cohorts. Of these, 4564 showed higher expression in the RLS cohort than in the PD cohort, whereas 3308 showed lower expression. These directions refer exclusively to the between-cohort comparison and not to changes relative to healthy individuals (Figure 1; Supplementary Table S1).

**Figure 2:**
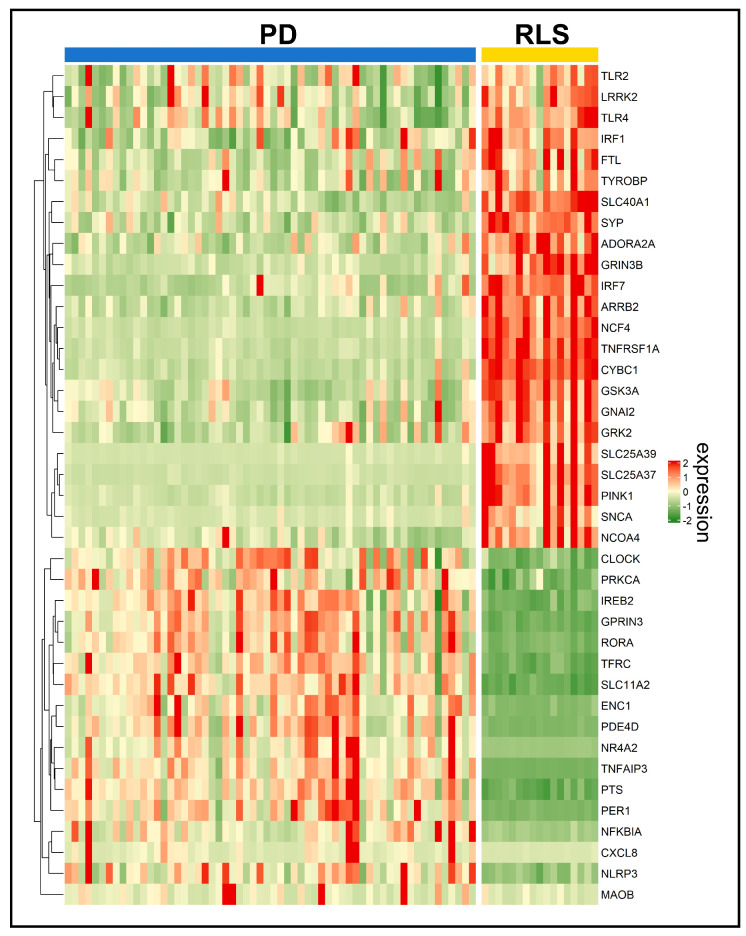
Expression heatmap of the hypothesis-guided 40-gene subset selected for focused visualization of the PD-versus-RLS between-cohort contrast.

To provide a focused visual and narrative summary of the between-cohort contrast, we assembled a hypothesis-guided subset of 40 genes from the complete set of genes meeting the differential-expression thresholds. The selected functional domains reflected the biological focus defined in the study objectives and the principal categories identified by pathway enrichment: iron homeostasis and ferroptosis; dopamine metabolism and tetrahydrobiopterin biology; GPCR-mediated signaling and synaptic regulation; neuroinflammation and oxidative stress; circadian regulation; and neuronal or mitochondrial homeostasis. Within these domains, genes were selected by jointly considering adjusted *p* value, absolute fold change, and expression level (baseMean) while representing both directions of the between-cohort contrast. This subset was used only for focused visualization and description in Table 1 and Figure 2 and should not be interpreted as an unbiased ranking of the 40 most significant genes. Differential-expression and pathway-enrichment analyses were conducted using the complete eligible gene set, reported in Supplementary Table S1.

Among the genes with relatively higher expression in the PD cohort than in the RLS cohort were several transcripts relevant to dopaminergic neuronal biology, dopamine metabolism, inflammatory regulation, circadian signaling, and iron transport, including *NR4A2*, *MAOB*, *PTS*, *ENC1*, *TFRC*, *IREB2*, *SLC11A2*, *NLRP3*, *PRKCA*, *CLOCK*, *RORA*, and *PER1*. The relatively higher expression of *NR4A2*, together with *MAOB* and *PTS*, defined a between-cohort pattern involving genes annotated to dopaminergic neuronal identity, dopamine handling, and tetrahydrobiopterin-dependent catecholamine biology. By contrast, genes with relatively higher expression in the RLS cohort than in the PD cohort included *SLC25A37*, *SLC25A39*, *SNCA*, *NCF4*, *TNFRSF1A*, *PINK1*, *IRF7*, *CYBC1*, *SLC40A1*, *SYP*, *FTL*, *NCOA4*, *LRRK2*, *TLR4*, *GNAI2*, *TLR2*, *IRF1*, and *TYROBP*. Within the hypothesis-guided subset, *ARRB2*, *GRK2*, *GSK3A*, *ADORA2A*, and *GRIN3B* also showed relatively higher expression in the RLS cohort and were annotated to dopaminergic and GPCR-associated signaling. Together, these genes represented pathway categories involving iron handling, ferroptosis-related biology, GPCR-mediated signaling, synaptic regulation, immune–redox processes, and mitochondrial quality control.

### 2.2. Ingenuity Pathway Analysis

**Figure 3:**
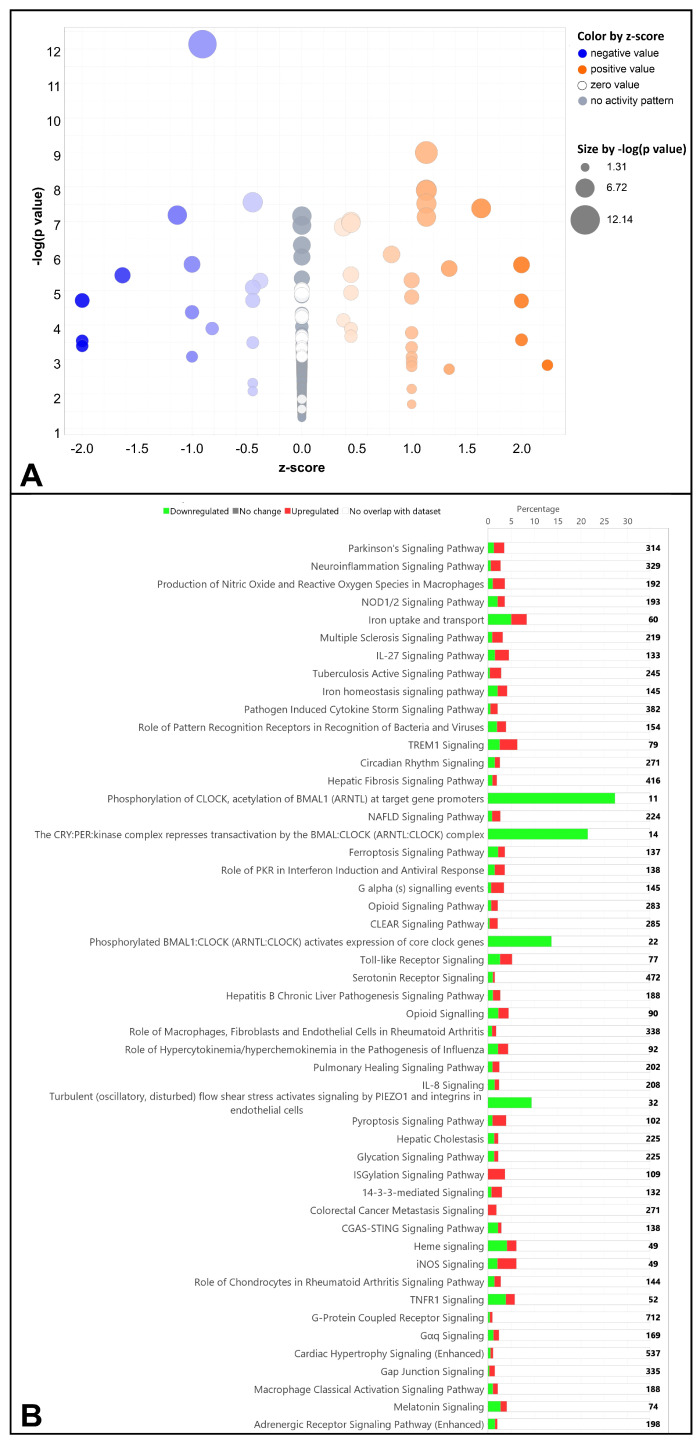
IPA canonical pathway enrichment analysis in RLS versus PD. (**A**) Volcano bubble plot of selected enriched pathways, with bubble size proportional to significance and color indicating activation z-score. (**B**) For each pathway category, the stacked bars indicate the percentages of genes showing relatively lower expression in the RLS cohort than in the PD cohort, no significant between-cohort difference, relatively higher expression in the RLS cohort than in the PD cohort, or no representation in the analyzed dataset. The IPA activation z-score represents a knowledge-base-derived prediction and should not be interpreted as a direct measurement of pathway activity or as evidence of tissue-specific activation.

Ingenuity Pathway Analysis identified significantly enriched canonical pathways (Figure 3A; Supplementary Table S2). The enriched pathways included Iron homeostasis signaling pathway, Iron uptake and transport, Ferroptosis Signaling Pathway, Production of Nitric Oxide and Reactive Oxygen Species (ROS), NRF2-mediated Oxidative Stress Response, Neuroinflammation Signaling Pathway, and Toll-like Receptor Signaling. Signaling cascades related to GPCR-mediated neurotransmission were also enriched, including Dopamine Receptor Signaling, Dopamine-DARPP32 Feedback in cAMP Signaling, cAMP-mediated signaling, Protein Kinase A Signaling, and GPCR Signaling. Pathways related to synaptic transmission and plasticity included Synaptic Long-Term Potentiation; Synaptic Long-Term Depression; and Glutamate binding, activation of AMPA receptors and synaptic plasticity.

Additional enriched pathways included GABAergic Receptor Signaling, Acetylcholine Receptor Signaling, Serotonin Receptor Signaling, Endocannabinoid Neuronal Synapse Pathway, Opioid Signaling, and Circadian Rhythm Signaling. Among the pathway categories with negative activation z-scores in the RLS-versus-PD contrast were Tetrahydrobiopterin Biosynthesis I, Tetrahydrobiopterin Biosynthesis II, and Parkinson’s Signaling Pathway. The relative proportions of genes showing higher expression in the RLS cohort than in the PD cohort, higher expression in the PD cohort than in the RLS cohort, no significant between-cohort difference, or no representation in the analyzed dataset are summarized in Figure 3B.

**Figure 4:**
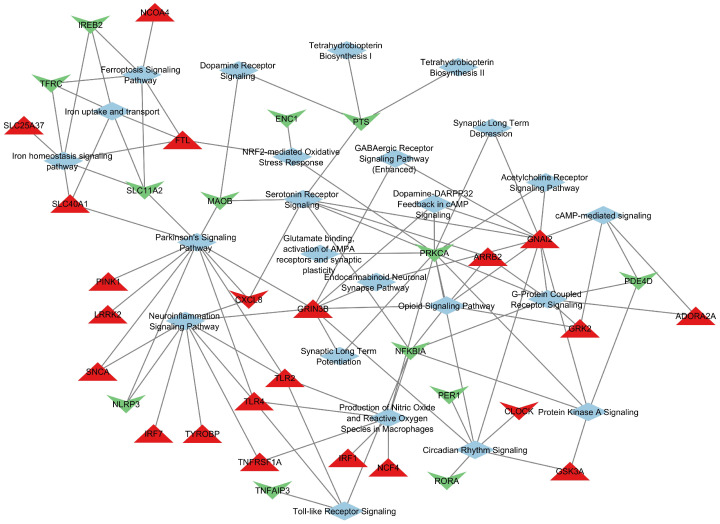
Cytoscape network of enriched canonical pathways and associated differentially expressed genes in the RLS-versus-PD between-cohort comparison. Nodes represent pathway categories or genes, and edges indicate gene–pathway relationships derived from Ingenuity Pathway Analysis. The network illustrates annotation overlap among iron homeostasis and ferroptosis, dopaminergic/GPCR signaling, synaptic regulation, inflammatory and oxidative-stress processes, and circadian-related pathways. It is intended as a descriptive visualization and should not be interpreted as evidence of causal, functional, or tissue-specific interactions.

Cytoscape network analysis further showed that the enriched pathways were organized into a few major interconnected modules (Figure 4). Iron-related pathways, including iron homeostasis signaling, iron uptake and transport, and ferroptosis signaling, were linked to genes such as *SLC25A37*, *SLC40A1*, *FTL*, and *NCOA4* while also connecting to *TFRC*, *IREB2*, and *SLC11A2*. A second densely connected module involved dopaminergic/GPCR-associated signaling, in which *GNAI2*, *ARRB2*, *GRK2*, *ADORA2A*, *PDE4D*, *PRKCA*, and *GSK3A* connected dopamine-DARPP32 feedback in cAMP signaling, cAMP-mediated signaling, GPCR signaling, and protein kinase A signaling. Pathways related to synaptic transmission and plasticity were connected mainly through *GRIN3B* and *PRKCA*, whereas circadian rhythm signaling was associated with *CLOCK*, *PER1*, and *RORA*. In parallel, Parkinson’s signaling, neuroinflammation, oxidative stress, and Toll-like receptor signaling were linked to genes including *SNCA*, *LRRK2*, *PINK1*, *TLR2*, *TLR4*, *TNFRSF1A*, *IRF1*, *IRF7*, *NCF4*, and *TYROBP*, highlighting the convergence of neurodegenerative, inflammatory, and redox-related processes in the overall network.

## 3. Discussion

This exploratory comparison identified broad differences in peripheral blood gene expression between the RLS and PD cohorts. The comparison remains clinically relevant because sleep disorders, including RLS and circadian disturbances, frequently intersect with PD [11], and dopaminergic mechanisms and symptomatic responsiveness are relevant to both disorders [14,15,36]. Within the present between-cohort contrast, relatively higher expression in the RLS cohort involved genes related to iron handling, GPCR-associated signaling, synaptic modulation, and immune-related pathways, whereas relatively higher expression in the PD cohort involved genes related to dopamine metabolism, dopaminergic neuronal identity, and Parkinson-related signaling. These broad biological domains are consistent with previous transcriptomic studies in RLS and PD [21,23,25]. However, because no healthy control group was included, the analysis cannot establish whether either profile is abnormal relative to a physiological baseline or represents a disease-specific alteration. The findings should therefore be interpreted only as relative differences between the two studied cohorts.

Within the present between-cohort comparison, several genes involved in iron homeostasis, transport, and ferroptosis, including *SLC25A37*, *SLC25A39*, *SLC40A1*, *FTL*, and *NCOA4*, showed relatively higher expression in the RLS cohort than in the PD cohort, whereas *TFRC*, *IREB2*, and *SLC11A2* showed relatively higher expression in the PD cohort. Iron-related biology was therefore represented on both sides of the contrast, but by different gene sets. This observation is compatible with the established relevance of altered iron handling and ferroptosis-related mechanisms to both disorders [3,21,22,30]. However, in the absence of healthy controls, it cannot determine which cohort deviates from physiological expression or establish disease-specific regulation of iron trafficking, storage, or cellular stress responses.

The relatively higher expression in the PD cohort of *TFRC*, *IREB2*, *SLC11A2*, and *NLRP3* is of interest because these genes are linked to iron transport, oxidative stress, and inflammasome-related processes implicated in Parkinsonian neurodegeneration. Conversely, *NCF4*, *CYBC1*, and several ferroptosis-related genes showed relatively higher expression in the RLS cohort. These patterns generate hypotheses concerning different peripheral representations of iron-, redox-, and ferroptosis-related processes but do not demonstrate iron accumulation, ferroptosis, or inflammasome activation in either disorder. Ferroptosis has been implicated in PD-related neurodegeneration [37] and more recently in RLS [22]. Similarly, the relatively higher expression of *NLRP3* in the PD cohort is biologically noteworthy in view of evidence linking α-synuclein to *NLRP3* activation [38], but it remains a relative peripheral expression difference rather than evidence of pathway activation.

*CYBC1*, *NCF4*, *TLR2*, *TLR4*, *IRF1*, *IRF7*, and *TYROBP* showed relatively higher expression in the RLS cohort than in the PD cohort and are annotated to innate immune, inflammatory, and Toll-like receptor-related processes. This between-cohort pattern is consistent with the literature implicating immune mechanisms in RLS [21,39,40], but it cannot establish neuroimmune activation in RLS or define disease-specific inflammatory profiles. Overall, the findings identify different relative representations of iron/ferroptosis- and immune-related genes in the two cohorts. The established biological and therapeutic relevance of iron to RLS is supported by previous evidence and current treatment guidelines [3,20,41], rather than demonstrated by the present comparison.

Within the present between-cohort comparison, relatively higher expression in the PD cohort involved genes annotated to dopaminergic neuronal biology and dopamine metabolism, including *NR4A2*, *PTS*, and *MAOB*, whereas relatively higher expression in the RLS cohort involved genes annotated to GPCR-mediated signaling and synaptic regulation, including *ARRB2*, *GRK2*, *GSK3A*, *ADORA2A*, *GNAI2*, and *GRIN3B*. These gene sets are relevant to striatal dopamine-related signaling; however, their differential expression in PBMCs does not demonstrate postsynaptic remodeling or a distinct form of central dopaminergic dysfunction.

Independent neuroimaging evidence provides some biological plausibility for considering limbic-striatal and mesolimbic mechanisms in RLS. Previous studies, including our structural MRI analysis, identified abnormalities involving interconnected limbic and striatal structures, while broader PET, SPECT, and MRI evidence has suggested involvement of both mesolimbic and nigrostriatal dopaminergic pathways in RLS [32,33]. The present peripheral findings may therefore generate hypotheses concerning mesolimbic-related mechanisms, but they cannot establish that the observed PBMC expression profile originates from, directly reflects, or identifies activity within these CNS circuits.

This interpretation should, however, be considered cautiously. The relatively higher expression in RLS than PD of genes such as *SNCA*, *SYP*, *LRRK2*, and *PINK1* may be relevant to synaptic vesicle dynamics, dopaminergic transmission, and mitochondrial quality-control pathways, but these findings do not demonstrate adaptive circuit plasticity or neuroprotective mechanisms [42]. Because these genes are also expressed in peripheral immune-cell populations, their relative expression may reflect differences in PBMC composition, medication exposure, or dataset structure rather than disease-specific neuronal mechanisms. Similarly, although some of these transcripts are involved in biological pathways relevant to mesolimbic dopaminergic signaling [43,44], and previous neuroimaging studies have suggested mesolimbic involvement in RLS [32,33], the present PBMC data cannot provide direct evidence of mesolimbic dopaminergic pathway involvement. At most, these findings generate hypotheses regarding neuromodulatory pathways that may contribute to the heterogeneous sensory phenotype of RLS, including painful and non-painful symptom dimensions [45,46].

By contrast, the present comparison showed that the following had relatively higher expression in PD than in RLS: *NR4A2* (*Nurr1*, an essential transcription factor for the development and maintenance of midbrain dopaminergic neurons); *PTS* and *MAOB*, both involved in dopamine biosynthesis and metabolism; and *ENC1*, which is linked to neuronal development. These genes are biologically relevant to dopaminergic neuronal identity and to pathways implicated in PD, including the nigrostriatal dopaminergic pathway [31]. However, because the present analysis was performed in PBMCs, this pattern should not be interpreted as direct evidence of nigrostriatal pathway activity or CNS localization. Rather, it identifies a relative peripheral transcriptomic difference between PD and RLS involving genes previously linked to dopaminergic biology and neurodegeneration [23,25]. The relatively lower expression of *NR4A2* in RLS than PD is noteworthy because it represents one of the strongest discriminating signals between the two groups.

Taken together, these data suggest that the peripheral transcriptomic contrast between RLS and PD involves different dopaminergic-related gene sets: relatively greater representation of signaling and network-modulation genes in RLS, and relatively greater representation of genes linked to dopaminergic neuronal identity, dopamine metabolism, and degeneration-related pathways in PD [36,47,48,49].

The clinical context motivating this comparison is the heterogeneity of RLS and RLS-like symptoms reported in PD. Similar manifestations may reflect true comorbid RLS, nocturnal motor restlessness, sensory discomfort, akathisia, dopaminergic treatment effects, or broader non-motor PD phenomena [10,11,12]. Genetic evidence also does not support a clear causal relationship or substantial shared genetic architecture between RLS and PD [13]. Within the limitations of the present study, the observed between-cohort differences raise the hypothesis that clinical overlap may coexist with different peripheral molecular profiles.

Dopaminergic agents may improve symptoms in both disorders but can also cause distinct long-term complications, including impulse-control disorders in PD and augmentation in RLS [14,15,17,18,19,20]. Nevertheless, the present findings do not support clinical classification, biomarker use, or therapeutic selection. Whether peripheral transcriptomic profiles have any value for PD phenotyping or treatment stratification can be determined only through independent replication and prospective validation in appropriately controlled cohorts.

As shown in Figure 3A,B, Ingenuity Pathway Analysis identified enrichment of gene sets annotated to multiple neurotransmitter, inflammatory, redox-related, iron-associated, and circadian processes. These findings indicate that genes assigned to these categories were statistically over-represented in the between-cohort contrast, but they do not by themselves demonstrate pathway activation or inhibition, causality, tissue-specific involvement, synaptic remodeling, or neuronal-circuit dysfunction. Nevertheless, independent CSF omics studies in RLS have identified alterations involving immune and inflammatory responses, coagulation, cytoskeletal regulation, hypoxia-related processes, and other molecular domains [50,51], while complementary CSF analyses have identified alterations in melanocortin and endorphin neuropeptides [5]. This partial convergence between peripheral and CNS-proximal findings supports the biological plausibility of some broad pathway domains represented in Figure 3, but it does not validate the specific genes, predicted pathway states, or CNS localization inferred from the present PBMC analysis. Accordingly, these findings remain hypothesis-generating and require confirmation in appropriately controlled cohorts and tissue-relevant experimental studies.

Figure 4 provides a visual organization of shared genes and annotation relationships among the enriched pathway categories shown in Figure 3. The network illustrates how individual genes are assigned to multiple functional categories and identifies potential points of pathway overlap. It should not, however, be interpreted as demonstrating functional interactions, coordinated systems-level reorganization, pathway activity, or CNS-specific molecular networks. Rather, it provides an exploratory framework for prioritizing genes and pathway categories for independent and functional validation.

Within the present between-cohort comparison, *CLOCK*, *PER1*, and *RORA* showed relatively lower expression in the RLS cohort than in the PD cohort, and Circadian Rhythm Signaling was among the enriched annotation categories. These findings identify a peripheral transcriptomic difference involving genes annotated to circadian-related processes. However, *CLOCK*, *PER1*, and *RORA* have pleiotropic roles beyond circadian regulation, and their expression in PBMCs may reflect peripheral clock function, immune regulation, or other cellular processes. The present results therefore cannot establish CNS circadian dysfunction, differences in neuronal-circuit involvement, or greater circadian dysregulation in either disorder. Nevertheless, the observation remains of potential interest because RLS has a well-established circadian symptom pattern and circadian disturbances are also clinically relevant in PD [52,53,54]. Its biological significance requires validation through studies incorporating controlled sampling times, repeated circadian measurements, and CNS-relevant or other tissue-specific approaches.

Overall, within this between-cohort comparison, relatively higher expression in the RLS cohort involved genes and pathways related to iron/ferroptosis biology, synaptic modulation, circadian-related signaling, and multisystem neurotransmitter pathways, whereas relatively higher expression in the PD cohort involved genes and pathways related to dopamine metabolism, tetrahydrobiopterin biosynthesis, inflammatory signaling, and oxidative-stress responses. These patterns describe only the direction of the PD-versus-RLS contrast. In the absence of healthy controls, they cannot be regarded as disease-specific abnormalities, used to determine which cohort deviates from physiological expression, or interpreted as establishing distinct disease mechanisms.

As increasingly emphasized in both clinical practice and research, a deeper understanding of the biological basis of PD is essential for identifying disease-modifying treatments capable of targeting the molecular underpinnings of the disorder [55]. Likewise, currently guideline-endorsed therapies for RLS are designed to modulate established pathophysiological mechanisms implicated in the disorder [20], underscoring the importance of identifying novel therapeutic targets grounded in molecular evidence [4].

These findings should also be interpreted in the context of the study design. Pathway-enrichment analysis identifies statistical over-representation of annotated gene sets but does not establish activation state, directionality, causality, tissue localization, or disease mechanism. In particular, pathway names referring to neuronal, synaptic, or neurotransmitter processes cannot be considered evidence of CNS involvement when derived from PBMC expression data. The present findings and the pathway patterns summarized in Figure 3 and Figure 4 should therefore be used only to generate hypotheses and prioritize candidate genes and pathways for validation in appropriately controlled cohorts, CNS-proximal samples, or biologically relevant experimental models.

The principal limitation of this study is that diagnostic group was completely confounded with dataset/batch because the two cohorts originated from independently generated datasets. Disease-related and technical effects therefore cannot be separated. The cohorts were also markedly unbalanced in size and differed substantially in age and sex distribution. Although age and sex were included as covariates in the differential-expression model, statistical adjustment may reduce but cannot eliminate their influence, particularly given the small RLS cohort, unequal group sizes, and limited demographic comparability. Residual age- and sex-related effects may therefore contribute to the observed transcriptomic contrast. Most participants with PD were also receiving dopaminergic therapy, whereas the RLS cohort was drug-free. Treatment may have influenced some findings, particularly dopamine-related and other treatment-sensitive pathways; however, its contribution cannot be quantified. The findings should therefore be interpreted only as an exploratory contrast between these specific cohorts and not as evidence of disease-specific molecular signatures. Independent replication in concurrently recruited, demographically matched cohorts processed within the same experimental workflow, ideally including untreated participants and healthy controls, is required. The use of PBMCs, the cross-sectional design, and the absence of functional validation further limit mechanistic and causal interpretation.

This exploratory analysis identified peripheral blood transcriptomic differences between the studied PD and RLS cohorts. Relative to the RLS cohort, the PD cohort showed higher expression of genes related to dopamine metabolism, tetrahydrobiopterin biology, oxidative stress, iron transport, and inflammatory signaling. Relative to the PD cohort, the RLS cohort showed higher expression of genes related to iron/ferroptosis biology, GPCR-associated signaling, synaptic modulation, and circadian-related pathways.

Because no healthy control group was included, neither profile can be classified as abnormal or disease-specific. Moreover, the complete confounding between diagnostic group and dataset/batch, together with demographic and treatment differences, prevents attribution of the observed contrast exclusively to diagnosis. These findings should therefore be regarded as hypothesis-generating observations from two specific cohorts.

Independent replication in concurrently recruited, demographically comparable cohorts processed within the same experimental workflow, including appropriate healthy controls and, ideally, untreated participants, will be required before the biological or clinical relevance of these differences can be established.

## 4. Materials and Methods

### 4.1. Study Design and Participants

This transcriptomic study included two patient groups. The RLS group comprised 17 drug-free patients (13 females and 4 males; mean age 55.8 years, range 24–76 years) of European ancestry. All patients had an International RLS Severity Scale [56] score > 15, indicating at least moderate symptom severity; the mean score was 21.1, and the mean disease duration was 6.6 years. RLS was diagnosed according to the International RLS Study Group criteria [1] by means of a semi-structured clinical interview and careful exclusion of RLS mimics. Exclusion criteria for the RLS group included: (a) a sleep disorder other than RLS; (b) psychiatric, neurologic, cardiovascular, or neurodegenerative disease; (c) neurodevelopmental delay; and (d) use of central nervous system (CNS) drugs within the year prior to the study or use of any drug or medication during the 3 weeks before polysomnographic recording. In addition, adult subjects with an apnea–hypopnea index greater than 10 events/hour of sleep were excluded. A family history of RLS was reported by two patients.

The PD group included 60 patients (39 males and 21 females) with a mean age of 68.6 years (range 37.4–87.0 years). PD was diagnosed clinically by expert neurologists at the time of enrollment, in agreement with the diagnostic criteria of the International Parkinson and Movement Disorder Society [7]. All participants with PD were assessed in the same hospital department by the same clinical staff involved in the evaluation of the RLS cohort. Screening for RLS symptoms is part of routine clinical assessment in this department, and none of the participants with PD reported RLS symptoms or fulfilled the diagnostic criteria for RLS. Mean disease duration was 4.6 years (median 3.0, range 0.5–20.0 years), mean Hoehn & Yahr (H&Y) stage [57] was 2.8 (range 1.0–5.0) and mean Mini-Mental State Examination (MMSE) score [58] was 26.1 (range 15–30). Forty-eight out of the 60 patients with PD were receiving dopaminergic treatment, and the mean levodopa equivalent daily dose (LEDD) [59] was 529.8 mg/day (range 26–1202 mg/day).

All procedures were performed in accordance with relevant laws, institutional guidelines, and the Declaration of Helsinki. The study protocol was approved by the Ethics Committee of the Oasi Research Institute-IRCCS, Troina, Italy. All participants provided written informed consent, and the privacy rights of participants were observed.

### 4.2. Blood Collection and PBMC Isolation

For all samples, venous blood was collected in the morning after a 12-h fast. Immediately after collection, samples were stratified using Ficoll-Paque PLUS (GE Healthcare Life Sciences, Piscataway, NJ, USA), and peripheral blood mononuclear cells (PBMCs) were stored at −80 °C until RNA isolation.

### 4.3. RNA Extraction

Total RNA was extracted from PBMCs using TRIzol Reagent (Invitrogen Life Technologies, Carlsbad, CA, USA), according to the manufacturer’s instructions. Samples were collected, stored, and processed using identical procedures. RNA extraction was performed simultaneously for samples within the same group, and isolated RNA was stored at −80 °C until further processing. A DNase treatment step was included after RNA isolation to ensure sample purity and accurate quantification. RNA yield and quality were assessed using a NanoDrop One spectrophotometer (Thermo Fisher Scientific, Waltham, MA, USA) and a TapeStation 4200 system (Agilent Technologies, Santa Clara, CA, USA), respectively.

### 4.4. Library Preparation and RNA Sequencing

RNA sequencing and primary data analysis were performed at Genomix4Life Srl (Baronissi, Italy). Indexed libraries were prepared from 600 ng of purified RNA using the Illumina Stranded mRNA Library Prep Kit (Illumina, Inc., San Diego, CA, USA). After enrichment of mRNA by oligo(dT) magnetic beads, purified mRNA was fragmented and reverse-transcribed into first-strand complementary DNA using random primers. During second-strand cDNA synthesis, dUTP replaced dTTP to preserve strand specificity. This was followed by adapter ligation and PCR amplification. Libraries were pooled in equimolar amounts and sequenced on the Illumina NovaSeq 6000 platform (Illumina, Inc., San Diego, CA, USA) using a 2 × 75 bp paired-end format.

### 4.5. Bioinformatic Processing

Raw sequencing files (FASTQ) underwent quality control using the FastQC tool (version 0.12.1) [60]. Low-quality reads, short reads (≤25 bp), and adapter sequences were removed using cutadapt (version 3.4) [61]. Filtered reads were aligned to the human reference genome using STAR (version 2.7.5c) [62] with standard parameters for paired-end reads. The reference genome corresponded to GENCODE HG38, Release 37 (GRCh38.p13). Gene-level quantification was performed using featureCounts (version 2.0.1) [63].

### 4.6. Differential Expression and Pathway Analysis

Gene expression data were analyzed in R (version 4.4.0) using the Bioconductor package DESeq2 (version 1.44.0) [64], which applies negative binomial generalized linear models. The differential-expression model included cohort, age, and sex as explanatory variables. Thus, the estimated between-cohort differences were adjusted for age and sex; however, this adjustment cannot fully overcome the marked demographic imbalance and limited sample size of the RLS cohort. Only genes expressed in at least 25% of samples were included in the analysis. *p* values were adjusted for multiple testing using the Benjamini–Hochberg method. Transcripts with a fold change ≥ 1.50 or ≤−1.50 (|FC| ≥ 1.50) and an adjusted *p* value ≤ 0.01 were considered differentially expressed. Heatmaps and volcano plots were generated using the ComplexHeatmap (v2.20.0) and EnhancedVolcano (version 1.22.0) in R (version 4.4.0), respectively.

After completion of the differential-expression and pathway-enrichment analyses, a hypothesis-guided subset of 40 genes was assembled for focused visualization and narrative description. Candidate genes were required to meet the study-wide differential-expression thresholds and to belong, according to IPA annotations and established gene functions, to biological domains corresponding to the study objectives or the principal enriched pathway categories. Within these domains, selection jointly considered adjusted *p* value, absolute fold change, and baseMean while including genes showing relatively higher expression in each cohort. This subset was not used to calculate differential expression or pathway enrichment, both of which were based on the complete eligible gene set.

Because the two groups were derived from independent datasets, dataset/batch and diagnostic group were completely confounded. Surrogate variable analysis was used only as an exploratory diagnostic approach to assess global sources of variation and cannot disentangle technical effects from diagnosis-related differences. Accordingly, the differential-expression results were interpreted conservatively as an exploratory between-cohort contrast, with dataset-related confounding considered a major structural limitation.

Ingenuity pathway analysis (IPA; QIAGEN, Redwood City, CA, USA) [65] was used to perform the functional and pathway analyses. In particular, canonical pathways were selected based on statistically significant (*p* value ≤ 0.01 and |FC| ≥ 1.50) coding genes. Cytoscape (version 3.10.3) [66] was used for the visualization of pathways and biological networks identified through Ingenuity Pathway Analysis (IPA), enabling the representation of complex networks. This software allows the construction and visualization of networks in which nodes represent genes or gene products, while edges represent the biological relationships between them. Advanced layout algorithms and visualization tools in Cytoscape were used to optimize the representation, facilitating the interpretation of interconnections.

## Figures and Tables

**Table 1:** Hypothesis-guided subset of differentially expressed genes selected for focused description of the PD-versus-RLS between-cohort contrast.

Gene	Base Mean	FC	log_2_ FC	log_2_ FC SE	*p*adj
*NR4A2*	515.193	−19.650	−4.296	0.197	<0.000001
*TNFAIP3*	5506.453	−9.267	−3.212	0.146	<0.000001
*CXCL8*	875.820	−8.751	−3.129	0.511	<0.000001
*PDE4D*	1228.113	−7.436	−2.895	0.156	<0.000001
*PER1*	3605.281	−7.164	−2.841	0.184	<0.000001
*ENC1*	829.397	−5.265	−2.396	0.169	<0.000001
*MAOB*	5.547	−4.351	−2.121	0.588	0.00077
*GPRIN3*	1752.004	−3.464	−1.793	0.096	<0.000001
*NFKBIA*	4863.240	−3.428	−1.777	0.219	<0.000001
*RORA*	2555.589	−3.313	−1.728	0.114	<0.000001
*PTS*	133.304	−3.207	−1.681	0.107	<0.000001
*TFRC*	957.549	−2.796	−1.483	0.094	<0.000001
*NLRP3*	688.280	−2.189	−1.130	0.152	<0.000001
*PRKCA*	720.069	−2.082	−1.058	0.106	<0.000001
*CLOCK*	334.671	−1.980	−0.985	0.106	<0.000001
*SLC11A2*	672.361	−1.954	−0.966	0.067	<0.000001
*IREB2*	1110.763	−1.856	−0.892	0.071	<0.000001
*TYROBP*	4134.613	1.541	0.624	0.091	<0.000001
*GRK2*	6816.530	1.574	0.654	0.056	<0.000001
*IRF1*	4527.105	1.640	0.714	0.136	<0.000001
*TLR2*	2904.097	1.672	0.741	0.144	<0.000001
*GNAI2*	8349.342	1.697	0.763	0.061	<0.000001
*TLR4*	1492.598	1.733	0.794	0.167	<0.000001
*LRRK2*	3187.318	1.794	0.843	0.170	<0.000001
*NCOA4*	5249.038	1.813	0.859	0.148	<0.000001
*FTL*	28,806.310	1.816	0.861	0.091	<0.000001
*GSK3A*	590.413	1.946	0.961	0.075	<0.000001
*ADORA2A*	17.205	2.089	1.063	0.194	<0.000001
*ARRB2*	6065.431	2.218	1.150	0.069	<0.000001
*SYP*	14.063	2.370	1.245	0.155	<0.000001
*SLC40A1*	558.795	2.674	1.419	0.152	<0.000001
*CYBC1*	1951.908	3.174	1.666	0.064	<0.000001
*IRF7*	33.397	3.854	1.946	0.343	<0.000001
*PINK1*	679.904	4.751	2.248	0.112	<0.000001
*TNFRSF1A*	1626.806	5.773	2.529	0.105	<0.000001
*NCF4*	514.692	6.728	2.750	0.119	<0.000001
*GRIN3B*	4.885	7.050	2.818	0.288	<0.000001
*SNCA*	779.393	11.999	3.585	0.333	<0.000001
*SLC25A39*	4444.459	16.348	4.031	0.213	<0.000001
*SLC25A37*	6794.369	16.659	4.058	0.171	<0.000001

FC = fold change; SE = standard error; *p*adj = Benjamini–Hochberg adjusted *p* value. The genes shown represent a hypothesis-guided descriptive subset and not an unbiased ranking of the 40 most statistically significant genes. Complete differential-expression results are provided in Supplementary Table S1.

## Data Availability

Raw sequencing data for the RLS cohort are publicly available through ArrayExpress/BioStudies under accession number E-MTAB-13155. Raw sequencing data for the PD cohort are publicly available through ArrayExpress/BioStudies under accession number E-MTAB-11326.

## Supplementary Materials

The following supporting information can be downloaded at: https://www.mdpi.com/article/10.3390/ijms27156576/s1.

## Abbreviations

The following abbreviations are used in this manuscript:

AMPA	α-amino-3-hydroxy-5-methyl-4-isoxazolepropionic acid
bp	base pairs
cAMP	cyclic adenosine monophosphate
cDNA	complementary DNA
CNS	central nervous system
D1	dopamine D1 receptor
D2	dopamine D2 receptor
D3	dopamine D3 receptor
D4	dopamine D4 receptor
DARPP32	dopamine- and cAMP-regulated phosphoprotein of 32 kDa
DESeq2	differential expression analysis for sequence count data 2
DNase	deoxyribonuclease
dTTP	deoxythymidine triphosphate
dUTP	deoxyuridine triphosphate
FASTQ	text-based format for nucleotide sequences and quality scores
FC	fold change
GABA	γ-aminobutyric acid
GENCODE	Encyclopedia of Genes and Gene Annotations
GPCR	G protein-coupled receptor
GRCh38	Genome Reference Consortium Human Build 38
H&Y	Hoehn and Yahr
HG38	human genome assembly GRCh38/hg38
IPA	Ingenuity Pathway Analysis
IRCCS	Istituto di Ricovero e Cura a Carattere Scientifico
LEDD	levodopa equivalent daily dose
MMSE	Mini-Mental State Examination
mRNA	messenger RNA
NRF2	nuclear factor erythroid 2-related factor 2
*p*adj	Benjamini–Hochberg adjusted p value
PBMC	peripheral blood mononuclear cell
PCR	polymerase chain reaction
PD	Parkinson’s disease
RNA	ribonucleic acid
RNA-seq	RNA sequencing
RLS	restless legs syndrome
ROS	reactive oxygen species
SE	standard error
STAR	Spliced Transcripts Alignment to a Reference
